# Crystallization and Alkaline Degradation Behaviors of Poly(l-Lactide)/4-Armed Poly(ε-Caprolactone)-Block-Poly(d-Lactide) Blends with Different Poly(d-Lactide) Block Lengths

**DOI:** 10.3390/polym12102195

**Published:** 2020-09-25

**Authors:** Suyang Dai, Min Wang, Zhuoxin Zhuang, Zhenbo Ning

**Affiliations:** State Key Laboratory of Organic-Inorganic Composites, Beijing Laboratory of Biomedical Materials, College of Life Science and Technology, Beijing University of Chemical Technology, Beijing 100029, China; 15010395008@163.com (S.D.); 15010930616@163.com (M.W.); miaozzx1997@163.com (Z.Z.)

**Keywords:** stereocomplex, crystallization, degradation, block length

## Abstract

Four-armed poly(ε-caprolactone)-*block*-poly(d-lactide) (4-C-D) copolymers with different poly(d-lactide) (PDLA) block lengths (*M_n,PDLA_*s) were synthesized by sequential ring-opening polymerization (ROP). The formation of stereocomplex (SC) crystallites in the 80/20 poly(l-lactide) (PLLA)/4-C-D blends were investigated with the change of *M_n,PDLA_* from 0.5 to 1.5 kg/mol. It was found that the crystallization and alkaline degradation of the blends were profoundly affected by the formed SC crystallites. The PLLA/4-C-D0.5 blend had the lowest crystallization rate of the three blends, and it was difficult to see spherulites in this blend by polarized optical microscopy (POM) observation after isothermal crystallization at 140 °C for 4 h. Meanwhile, when *M_n,PDLA_* was 1 kg/mol or 1.5 kg/mol, SC crystallites could be formed in the PLLA/4-C-D blend and acted as nucleators for the crystallization of PLLA homo-crystals. However, the overall crystallization rates of the two blends were still lower than that of the neat PLLA. In the PLLA/4-C-D1.5 blend, the Raman results showed that small isolated SC spherulites were trapped inside the big PLLA homo-spherulites during isothermal crystallization. The degradation rate of the PLLA/4-C-D blend decreased when *M_n,PDLA_* increased from 0.5 to 1.5 kg/mol, and the degradation morphologies had a close relationship with the crystallization state of the blends. This work revealed the gradual formation of SC crystallites with the increase in *M_n,PDLA_* in the PLLA/4-C-D blends and its significant effect on the crystallization and degradation behaviors of the blend films.

## 1. Introduction

Biodegradable polymers have received widespread attention due to increasing environmental concern [1,2,3]. Among them, polylactic acid (PLA) has been widely used in packaging and biomedical fields [4]. It is categorized into three forms: poly(l-lactide) (PLLA), poly(d-lactide) (PDLA), and poly(d,l-lactide) (PDLLA). Commercial PLA is usually an l-rich mixture as the majority of bacteria used in fermentation processes predominantly produce l-lactic acid [5]. However, the lower ability of crystallization, poor thermal stability and brittleness of PLLA limit its further application [6,7]. As a result, various methods have been used for the modification of PLLA [8].

Poly(ε-caprolactone) (PCL) is also a famous degradable polymer with excellent biocompatibility, biodegradability, and it is very flexible at room temperature because of a very low glass transition temperature (*T*_g_) of about −60 °C [9,10]. As a result, it is considered as a reasonable modifier to improve the ductility of the PLLA matrix by melt-blending [11]. However, it was found that the poor interfacial compatibility between PLLA and PCL limits the enhancement of the mechanical properties [12,13]. The copolymer of PCL and PLA can enhance the interfacial adhesion between the two phases and is used as a compatibilizer for the PLLA/PCL blend [14]. In recent years, copolymers or composites with soft middle blocks and outer PDLA blocks have been a hotspot of scientific interest as PLLA modifiers [7,15,16]. In these modifiers, the soft middle segments could be a PCL copolymer and introduce elastic properties into the material [17]. Meanwhile, the outer segments are PDLA chains, which can form stereocomplex (SC) crystallites with the PLLA matrix and act as physical crosslinking points in the blends. The soft and hard segments would have a synergetic effect on the mechanical property of PLLA and could improve its toughness effectively. SC crystallites have a higher melting temperature than neat PLLA, and they could accelerate the crystallization of PLLA homo-crystallites by acting as nucleators [18,19,20]. However, the formation mechanism of the SC crystallites in the blends of the “soft–hard” modifiers and PLLA need to be further investigated, in particular, the effect of the PDLA block length on the properties of the blend has not been fully elucidated yet.

In this work, we synthesized a series of four-armed poly(ε-caprolactone)-*block*-poly(d-lactide) copolymers (4-C-D) with different PDLA block lengths (*M_n,PDLA_*: 0.5, 1 and 1.5 kg/mol). The gradual formation of SC crystallites in these blends with increasing *M_n,PDLA_* was investigated. The reason for choosing *M_n,PDLA_* as 0.5, 1, 1.5 kg/mol is that the shortest block length of PDLA for stereocomplexation was in the range of 0.5–1.5 kg/mol, and it is interesting to study the crystallization and degradation behavior of the blends when *M_n,PDLA_* is around the critical block length. The block copolymer was denoted as 4-C-Dx, where x represents the block length of the PDLA (*M_n,PDLA_*). In these copolymers, PCL with a fixed block length of 1 kg/mol was used as the soft segments, and PDLA with different block lengths acted as the hard segments for the modification of PLLA. The crystallization mechanism of the PLLA/4-C-D blends, especially the effect of *M_n,PDLA_* on the stereocomplexation will be discussed. Besides, the relationship of the crystallization state and the degradation behavior of the blend films was also investigated.

## 2. Materials and Methods

### 2.1. Materials

l-lactide (Purasorb L, LLA) and d-lactide (Purasorb D, DLA) were purchased from Purac Biochem (Gorinchem, the Netherlands) and purified by recrystallization from ethyl acetate three times. ε-caprolactone (CL, 99%) and ethylene glycol (Analytical Reagent grade, ≥99.8%) were purchased from Sigma-Aldrich Chemicals (Shanghai, China) and Sinopharm Chemical Reagent Beijing Co., Ltd. (Beijing, China), respectively. They were dried with calcium hydride (CaH_2_) and distilled under reduced pressure. Toluene (Analytical Reagent grade, ≥99.5%) was purchased from Sinopharm Chemical Reagent Beijing Co., Ltd. and was distilled in the presence of metallic sodium and benzophenone. Stannous octoate (Sn(Oct)_2_) (Sigma-Aldrich, Shanghai, China) was dissolved in dehydrated toluene under the protection of argon. Pentaerythritol (Sigma-Aldrich, Shanghai, China) was used as purchased.

### 2.2. Methods

#### 2.2.1. Synthesis of 4-C-D Copolymers

4-C-D copolymers with a fixed PCL block length and different PDLA block lengths were prepared by sequential ring-opening polymerization (ROP) using pentaerythritol as the initiator. A representative procedure is described briefly as follows: a clean dry Schlenk flask was degassed and purged three times with argon, then the desired amounts of CL and pentaerythritol were added into the flask under an argon atmosphere. The solution polymerization in toluene proceeded at 110 °C for 48 h upon addition of Sn(Oct)_2_. After that, the desired amount of d-lactide (DLA) was introduced into the flask under the argon atmosphere and the polymerization was carried out for another 48 h. The block lengths of PCL and PDLA were controlled by varying the molar ratio of CL and DLA to pentaerythritol. PLLA was synthesized by using ethylene glycol as the initiator with a similar process. The synthesized polymers were purified by repeated precipitation using dichloromethane as a solvent and methyl alcohol as a nonsolvent. The products were dried in vacuo at 40 °C for 2 days before use.

#### 2.2.2. Sample Preparation

For the preparation of PLLA/4-C-D blends, certain amounts of 4-C-D and PLLA were first dissolved in chloroform at room temperature (25 °C) to obtain solutions with a concentration of 20 mg/mL with vigorous stirring for 24 h. Then the mixed solution was cast onto a Petri dish and evaporated at 25 °C for 2 days. The obtained films were dried in vacuo for another 2 days at 40 °C to remove the residual solvent. The mixing ratios of PLLA/4-C-D were fixed at 8/2 (*w/w*).

#### 2.2.3. Analysis and Characterization

The ^1^H NMR spectra of the polymerization products were recorded on a Varian 400 MHz spectrometer at room temperature with CDCl_3_ as a solvent.

The relative molecular weights of the synthesized polymers were measured with a Waters 1515 gel permeation chromatography (GPC) system. The GPC measurement was carried out at 40 °C with THF as the eluent at a flow rate of 1.0 mL/min, and polystyrene was used as the standard.

Wide-angle X-ray diffraction (WAXD) data were acquired at a scan speed of 5 °/min using a Rigaku diffractometer with Cu Kα radiation (wavelength λ = 0.154 nm) operating at 40 kV and 110 mA in the 2θ range from 5° to 40°. The film samples for WAXD measurement were compressed into sheets by a hot compression molding machine. Briefly, a certain amount of blend was first sandwiched between two Teflon sheets with another Teflon sheet (0.15 mm thickness) as a spacer. Then the sandwiched films were compressed at 225 °C with a pressure of 5 MPa for 3 min. After that, the films were transferred rapidly onto a hot plate at 140 °C and isothermally crystallized for 8 h.

Differential scanning calorimetry (DSC) measurements of the samples (approximately 5 mg) were conducted using a DSC-Q2000 (TA Instruments, New Castle, DE, USA) within the temperature range of −65 to 225 °C. The calibration was performed with indium and hexatriacontane and all the tests were carried out under a nitrogen atmosphere. To determine the glass transition temperature (*T_g_*), the samples were first melted at 225 °C for 3 min, and then quenched to 0 °C. After holding at 0 °C for 3 min, the samples were then heated to 100 °C at a rate of 5 °C min^−1^. For isothermal crystallization, after erasing thermal history at 225 °C, the samples were quenched to 140 °C to crystallize for enough time and subsequently heated to 225 °C at 10 °C/min.

The spherulite morphology of the crystallized samples was observed by a polarized optical microscope (POM, Leica DMLP, Wetzlar, Germany) equipped with a camera system and a Linkam THMS-600 hot stage. Certain amounts of PLLA and 4-C-D were first dissolved in chloroform at room temperature (25 °C) and stirring for 24h, followed by a solvent evaporation process on glass substrate to obtain films for POM observation.

The same method used in the WAXD test was employed to prepare the samples for alkaline degradation which was carried out at 37 °C in NaOH solution (1 M). The surface morphology of the films after alkaline degradation was characterized by using a scanning electron microscope (SEM, Fei Inspect FEI, Eindhoven, the Netherlands) with an accelerating voltage of 5.0 kV. All the samples were sputter-coated with a thin layer of gold.

## 3. Results and Discussion

### 3.1. Synthesis and Characterization of 4-C-D

**Figure 1:**
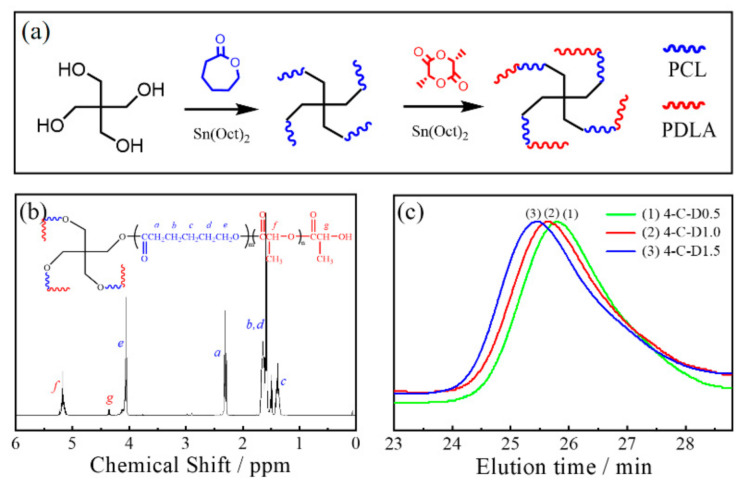
Synthesis of the 4-C-D copolymers with different *M_n,PDLA_*s. (**a**) Synthesis scheme, (**b**) ^1^H NMR spectrum, and (**c**) GPC curves.

The 4-C-D copolymers were synthesized by sequential ROP, as shown in Figure 1a. The ^1^H NMR spectrum and GPC curves of the copolymers are shown in Figure 1b,c and the molecular weights are summarized in Table 1. The signals at 4.3 ppm (f) and 5.2 ppm (g) were assigned to the CH_3_ and CH in the PDLA block, while the signals at 1.39 (c), 1.66 (b,d), 2.31 (a), and 4.1 (e) ppm were assigned to the methylene protons (CH_2_CH_2_CH_2_CH_2_CH_2_) of the PCL block. The GPC traces in Figure 1c show that each copolymer has a monomodal elution peak, and these peaks shift gradually to a shorter elution time region with increasing *M_n,PDLA_*. Moreover, the polydispersity index of each copolymer (*M_w_/M_n_*) is lower than 1.2 (Table 1), indicating the good control of the molecular weights of the four-armed copolymers by the sequential ROP method.

### 3.2. Miscibility of the PLLA/4-C-D Blends

It is well known that the glass transition temperature (*T_g_*) of a polymer material determines its crystallization behavior [21]. The neat PLLA showed a single *T_g_* at around 59.5 °C, while the *T_g_* of PLLA/4-C-D0.5 decreased sharply to around 44.0 °C, indicating the increased mobility of PLLA chains as a result of the 4-C-D modifier in the blend. The *T_g_* values for PLLA/4-C-D1 and PLLA/4-C-D1.5 increased to 45.9 and 48.3 °C, respectively, which could be attributed to the decreasing content of PCL components with the increase in *M_n,PDLA_*.

### 3.3. Crystal Structure of the PLLA/4-C-D Blends

**Figure 2:**
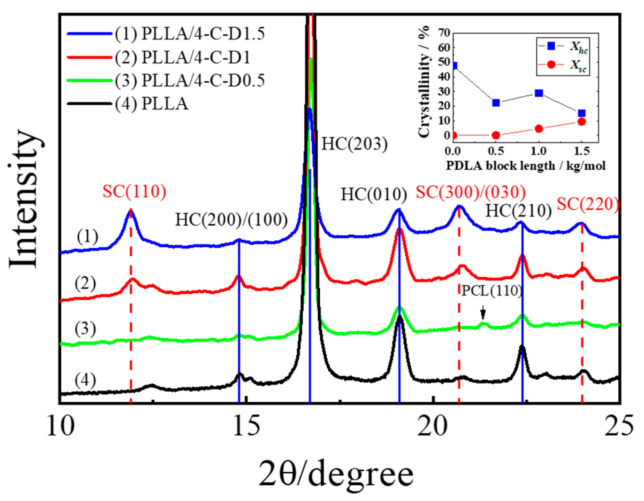
WAXD curves and crystallinities of PLLA and the PLLA/4-C-D blends with different PDLA block lengths after isothermal crystallization at 140 °C for 8 h.

To investigate the effect of PDLA block length on the crystal structure of the PLLA/4-C-D blends, the WAXD measurements were carried out and the obtained patterns are shown in Figure 2. The diffraction peaks at 2θ ≈ 12.5°, 14.9°, 16.9°, 19.3°, and 22.3°, which correspond to the (103), (010), (200)/(110), (203), and (210) planes of the α-form crystals of PLLA [22], could be observed in all the samples. Meanwhile, the diffraction peaks at 2θ ≈ 11.9°, 20.6°, and 23.8° correspond to the (110), (300)/(030), and (220) planes of the SC crystals, respectively [23]. The three peaks of the SC crystallites are present in the PLLA/4-C-D1, PLLA/4-C-D1.5, and PLLA/4-C-D2 blends.

In the PLLA/4-C-D0.5 blend, the diffraction peak at 2θ ≈ 21.3° for (200) plane of PCL crystals could be observed and it disappeared when *M_n,PDLA_* was higher, which means the crystallization of the PCL was inhibited significantly as the *M_n,PDLA_* increased. No SC crystallites were formed when *M_n,PDLA_* was 0.5 kg/mol, and it should be noted that *M_n,PDLA_* was longer than the minimum block length of PDLA needed to form SC crystallites in the PLLA/PDLA blend reported by Hennink et al. [24]. This means the stereocomplexation was affected by the chemical structure of the star-shaped block polymer, and a longer PDLA block length was needed in the copolymer to form SC crystallites with the PLLA matrix.

The crystallinities of the blend were calculated using the following equations:

(1) $$X_{\mathrm{sc}}\;=\;\frac{I_{hc}}{I_{total}}\times 100\%$$

(2) $$X_{\mathrm{sc}}\;=\;\frac{I_{sc}}{I_{total}}\times 100\%$$

*I_total_*, *I_h_*, and *I_sc_* are the total diffraction area, diffraction area of the homo-crystallites, and SC crystallites in the WAXD pattern, respectively. Meanwhile, *X_hc_* and *X_sc_* represent the crystallinities of PLLA homo-crystals and SC crystals, respectively. It is shown in Figure 2 that the *X_hc_* for PLLA was 47.6%, and it decreased a lot to 22.2% when the 4-C-D0.5 copolymer was introduced into PLLA. Then it increased to 28.8% as SC crystallites (*X_sc_* = 4.5%) were formed in the PLLA/4-C-D1 blend, which acted as nucleation sites for PLLA homocrystallization. The *X_hc_* decreased to 15.0% with an increased *X_sc_* (9.4%) in the PLLA/4-C-D1.5 blend, indicating that there was competition between homocrystallization and stereocomplexation.

### 3.4. Spherulite Morphologies and Growth Rates

**Figure 3:**
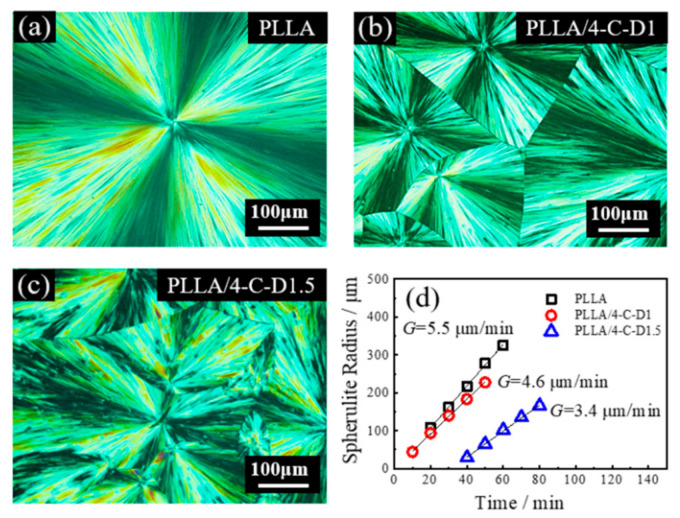
Spherulite morphologies and radial growth rates for PLLA and the PLLA/4-C-D blends. (**a**) PLLA, (**b**) PLLA/4-C-D1, (**c**) PLLA/4-C-D1.5, and (**d**) radial growth rates at 140 °C.

The POM observation was performed to investigate the crystallization morphologies of the PLLA/4-C-D blends, and the results are shown in Figure 3. It can be seen that PLLA showed distinct spherulites with well-defined Maltese crosses. Meanwhile, no spherulite could be seen in the PLLA/4-C-D0.5 blend even after isothermal crystallization for 4 h, revealing that PLLA crystallization was depressed profoundly in the blend because of the dilution effect of the 4-C-D0.5 copolymer. In the PLLA/4-C-D1 (Figure 3b) or PLLA/4-C-D1.5 blend (Figure 3c), the spherulite number per unit area increased compared to pure PLLA. From the WAXD results in Figure 2, we know that SC crystallites could be formed in the two blends, so it means they acted as nucleation sites for PLLA homocrystallization. Plots of spherulite radius versus time are presented in Figure 3d. It can be seen that the neat PLLA exhibited the highest *G* value of 5.5 μm/min, and it decreased to 4.6 μm/min in the PLLA/4-C-D1 blend. When the PDLA block length increased to 1.5 kg/mol, the *G* value decreased further to 3.4 μm/min.

**Figure 4:**
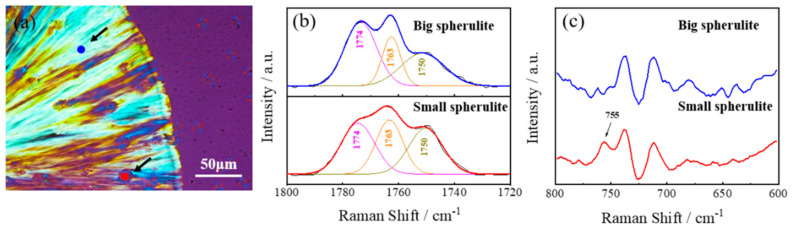
(**a**) Certain areas of the big (blue dot) and small spherulites (red dot) in the PLLA/4-C-D1.5 blend. (**b**) Raman spectra in the range of 1720–1800 cm^−1^ and the peak-fitting result within the νC=O region. (**c**) Raman spectra in the range of 600–800 cm^−1^.

It should be noted that as the PDLA block length increased to 1.5 kg/mol in the PLLA/4-C-D1.5 blend, and small isolated spherulites were seen trapped inside the big spherulites. The enlarged picture of this blend is shown in Figure 4a. The Raman measurement was employed to further investigate the crystal structure of the different spherulites. Figure 4b,c show the Raman spectra at certain areas (represented by blue and red dots in Figure 4a) of the big spherulite region and small spherulite region, respectively. Figure 4b illustrates the peak-fitting result within the ν_C=O_ region and three peaks are applied. A similar fitting process was used as reported in [25]. The Raman spectra in the range of 600–800 cm^−1^ are also shown in Figure 4c. Zhang et al. reported that SC crystallites had a much stronger band at around 1749 cm^−1^ and a band at around 754 cm^−1^. In our study, it was found that the Raman spectrum of the small spherulite region (red dot in Figure 4a) had a clear band at 755 cm^−1^ and it had a stronger band at around 1750 cm^−1^, while no signal was found at 755 cm^−1^ for the big spherulite region (blue dot) and it had a weaker band at 1750 cm^−1^ compared to the small spherulite. These results mean that the small spherulites in the blend should be attributed to the SC crystallites, while the big spherulites should attributed to the PLLA homo-crystallites.

### 3.5. Isothermal Crystallization

**Figure 5:**
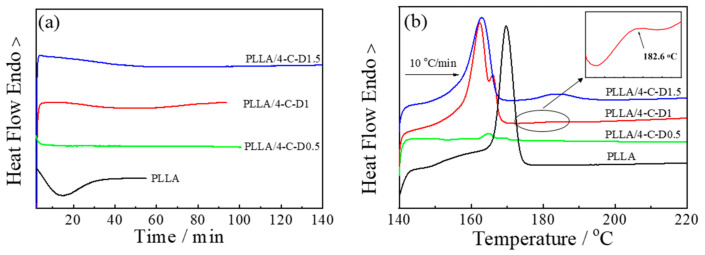
Isothermal (**a**) and heating (**b**) DSC curves of PLLA and PLLA/4-C-D blends with PDLA block lengths.

Figure 5 shows the DSC isothermal thermograms and the subsequential heating curves of PLLA and PLLA/4-C-D blends, and the thermal properties are listed in Table 2. It was found that the crystallization rates of all the blend samples were slower than that of the neat PLLA during isothermal crystallization at 140 °C, shown in Figure 5a, revealing that the crystallization of PLLA was retarded by the 4-C-D copolymers.

The effect of star-shaped copolymers seems to be different compared to that in our earlier work [16], in which the rate of PLLA crystallization was accelerated by the addition of star-shaped block copolymers. Actually, the temperatures to remove the thermal history in the two papers were different. In the earlier work, the temperature was 180 °C, which means the SC crystallites were not melted in the blends and could act as nucleators effectively during isothermal crystallization. However, in the present work, this temperature was 225 °C, which was higher than the melting temperature of the SC crystallites, which means that the SC crystallites needed to be formed and could not affect the crystallization of the blend as in the earlier work.

When PLLA was blended with 4-C-D0.5, no exothermic peak was observed within 100 min of the isothermal process, revealing that the crystallization rate was depressed significantly by the dilution effect of 4-C-D0.5. In the PLLA/4-C-D1 blend, the *t_p_* value was 47.6 min, indicating that the crystallization rate of this blend was faster than that of the PLLA/4-C-D0.5 blend, but it was still slower than that of the pure PLLA. These results were consistent with Figure 3c,d, showing that although the SC crystallites could be formed and act as nucleation sites for PLLA homocrystallization in this blend, the dilution effect of the melting PCL on the amorphous PLLA might still depress PLLA crystallization significantly, causing a slower crystallization rate than the neat PLLA sample. When increasing *M_n,PDLA_* to 1.5 kg/mol in the PLLA/4-C-D1.5 blend, the *t_p_* value increased to 72.3 min, revealing that competition between PLLA homocrystallization and stereocomplexation might exist in the blend, which could retard PLLA homocrystallization during the isothermal process.

Figure 5b shows the heating curves of all the samples after isothermal crystallization. It shows that the PLLA/4-C-D1 and PLLA/4-C-D1.5 blends exhibited two kind of endothermal peaks. The peaks in the range of 170–195 °C were attributed to the melting of SC crystals, while peaks in the range of 150–170 °C were attributed to the melting of PLLA homo-crystals. The melting temperatures of homo-crystals (*T_m,hc_*) in the blends were lower than in neat PLLA, revealing the disturbed structures of PLLA homo-crystallites in the blends. No melting peaks of SC crystallites were seen in the PLLA/4-C-D0.5 blend, which was consistent with the WAXD results in Figure 2. Besides, the competition between homocrystallization and stereocomplexation was confirmed by the increasing *X_sc_* and decreasing *X_hc_* with the increasing PDLA block length.

### 3.6. Degradation Behavior

**Figure 6:**
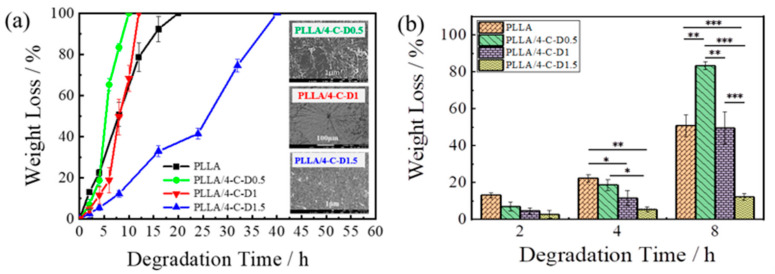
(**a**) Weight loss and degradation morphologies of the PLLA/4-C-D blend films with different PDLA block lengths. (**b**) Weight loss of PLLA/4-C-D blend films after degradation for 2, 4 and 8 h (* *p* < 0.05, ** *p* < 0.01, *** *p* < 0.001, *n* = 3).

It is well known that the degradation of PCL and PLA are carried out through the cleavage of ester bonds [26], and the degradation behavior of degradable polymers has a close relationship with the crystalline state [27]. The films were prepared by the same process as that in the WAXD measurements. The weight loss of the PLLA/4-C-D blend films during alkaline degradation is shown in Figure 6a. Statistical analysis of the weight loss by one-way ANOVA was performed for PLLA and the PLLA/4-C-D blend films after degradation for 2, 4, and 8 h and is shown in Figure 6b. Quantitative results were obtained from triplicates. It can be seen that the degradation rate of the PLLA/4-C-D blend film decreased with growing PDLA block length. The reason for the slower degradation rate of the PLLA/4-C-D1 blend film compared to that of the PLLA/4-C-D0.5 blend film was that the *X_hc_* (28.8%) increased when the SC crystallites were formed and acted as nucleators for PLLA homocrystallization in the blend. In the PLLA/4-C-D1.5 blend film, the *X_hc_* decreased to 15.0% while the *X_sc_* increased to 9.4%. It is known that the SC crystallites have a slower hydrolytic degradation rate than neat PLLA [28] and, as a result, the degradation rate decreased with the increased SC crystallites in the PLLA/4-C-D1.5 blend. The degradation morphologies after degradation for 4 h are also shown in Figure 6a. The surface of the PLLA/4-C-D0.5 blend film showed the largest spherulite morphologies of the three blends. In the PLLA/4-C-D1 blend, much smaller spherulite structures were seen after degradation compared to those in the PLLA/4-C-D0.5 blend film. However, no clear spherulite structures were seen in the PLLA/4-C-D1.5 blend film, revealing that it had a dense structure because of the SC crystallites in the blend.

## 4. Conclusions

A series of 4-C-D with different *M_n,PDLA_*s (0.5, 1, 1.5 kg/mol) was successfully prepared by ring-opening polymerization. The results of ^1^H NMR and GPC indicated that the block length of each component in the copolymers was well controlled. WAXD results showed that SC crystallites could be formed when *M_n,PDLA_* increased to 1 kg/mol, however, no clear morphologies of SC crystallites were found in the POM pictures, indicating that they acted mainly as nucleators for PLLA homo-crystallites. In the PLLA/4-C-D1.5 blend, small SC crystallites were trapped in the big spherulites of PLLA homo-crystals. The growth rates of the homo-crystalline spherulites in the PLLA/4-C-D blends were slower than that of the neat PLLA and decreased with the increasing of *M_n,PDLA_*. Although the *T_g_* results revealed that the melting PCL component could enhance the mobility of PLLA chains, it would also cause the dilution effect and depress PLLA crystallization in the blend. The SC crystallites in the PLLA/4-C-D1 blend could accelerate the crystallization rate of PLLA compared to the PLLA/4-C-D0.5 blend, however, a competition between stereocomplexation and homocrystallization would retard PLLA crystallization in the PLLA/4-C-D1.5 blend. In alkaline degradation, the degradation rate of the PLLA/4-C-D blend decreased as *M_n,PDLA_* increased. The spherulites in the PLLA/4-C-D1 blend after degradation for 4 h were much smaller than those in the PLLA/4-C-D0.5 blend. The PLLA/4-C-D1.5 blend film was much harder to degraded and no clear spherulite structures were seen in the film, indicating that the resistance to alkaline degradation was enhanced a lot as a result of the increased SC crystallites.

## Figures and Tables

**Table 1:** Characteristics of PLLA and 4-C-D copolymers with different *M_n,PDLA_*s.

Sample	*M_n,PCL_*^(a)^ (g/mol)	*M_n,PDLA_*^(a)^ (g/mol)	*M_n,NMR_*^(b)^ (g/mol)	*M_n,GPC_*^(c)^ (kg/mol)	*M_w,GPC_*^(c)^ (kg/mol)	*PDI* ^(c)^	*Conversion* (%)
PLLA	-	-	-	49.1	71.2	1.45	95.3
4-C-D0.5	1381	513	7715	8.3	10.3	1.16	78.4
4-C-D1	911	876	7283	10.9	13.0	1.19	81.8
4-C-D1.5	897	1360	9163	14.0	16.1	1.15	84.1

^(a)^*M_n,PCL_* and *M_n,PDLA_* are the block lengths of PCL and PDLA in the 4-C-D copolymer determined from the ^1^H NMR results. ^(b)^ *M_n,NMR_* is the number-averaged molecular weight determined from the ^1^H NMR results. ^(c)^ Number-averaged molecular weight (*M_n,GPC_*), weight-averaged molecular weight (*M_w,GPC_*), and molecular weight distribution (*PDI*) were measured from GPC analysis in tetrahydrofuran with narrow-distributed polystyrenes as standards.

**Table 2:** Characteristic values of isothermal crystallization and subsequential heating process of PLLA and its blends with different PDLA block lengths.

Sample	*t_p_*^ (a)^ (min)	*T_m,hc_*^ (b)^ (°C)	*T_m,sc_*^ (b)^ (°C)	Δ*H_m,hc_*^ (c)^ (J/g)	Δ*H_m,sc_* ^ (c)^ (J/g)	*X_c,hc_*^ (d)^ (%)	*X_c,sc_*^ (e)^ (%)
PLLA	15.0	170.0	-	42.7	-	45.9	-
PLLA/4-C-D0.5	-	164.8, 169.7	-	1.73	-	1.9	-
PLLA/4-C-D1	47.6	162.4, 168.4	182.6	46.3	0.44	49.8	0.78
PLLA/4-C-D1.5	72.3	163.0	184.3	41.4	4.42	44.5	7.78

^(a)^*t_p_* is the crystallization peak time during isothermal crystallization. ^(b)^ *T_m,hc_* and *T_m,sc_* are the melting temperatures of homo-crystallites and SC crystallites, respectively. ^(c)^ Δ*H_m,hc_* and Δ*H_m,sc_* are the fusion enthalpies of homo-crystallites and SC crystallites, respectively. ^(d)^ *X_hc_* = Δ*H_m,hc_/*${\Delta H}_{m,\mathrm{hc}}^{0}\;\times$ 100%, ${\Delta H}_{m,\mathrm{hc}}^{0}$ = 93 J/g. ^(e)^ *X_sc_* = Δ*H_m,sc_/*${\Delta H}_{m,\mathrm{sc}}^{0}\;\times$ 100%, ${\Delta H}_{m,\mathrm{sc}}^{0}$ = 142 J/g.

## References

[1] Tyler B., Gullotti D., Mangraviti A., Utsuki T., Brem H. (2016). Polylactic acid (PLA) controlled delivery carriers for biomedical applications. Adv. Drug. Deliv. Rev..

[2] Amass W., Amass A., Tighe B. (1998). A review of biodegradable polymers: Uses, current developments in the synthesis and characterization of biodegradable polyesters, blends of biodegradable polymers and recent advances in biodegradation studies. Polym. Int..

[3] Shi X.D., Sun P.J., Gan Z.H. (2018). Preparation of porous polylactide microspheres and their application in tissue engineering. Chin. J. Polym. Sci..

[4] Rasal R.M., Janorkar A.V., Hirt D.E. (2010). Poly(lactic acid) modifications. Prog. Polym. Sci..

[5] Saeidlou S., Huneault M.A., Li H.B., Park C.B. (2012). Poly(lactic acid) crystallization. Prog. Polym. Sci..

[6] Xu H., Teng C.Q., Yu M.H. (2006). Improvements of thermal property and crystallization behavior of PLLA based multiblock copolymer by forming stereocomplex with PDLA oligomer. Polymer.

[7] Li Z.B., Muiruri J.K., Thitsartarn W., Zhang X., Tan B.H., He C.B. (2018). Biodegradable silica rubber core-shell nanoparticles and their stereocomplex for efficient PLA toughening. Compos. Sci. Technol..

[8] Yang Y., Zhang L.S., Xiong Z., Tang Z.B., Zhang R.Y., Zhu J. (2016). Research progress in the heat resistance, toughening and filling modification of PLA. Sci. China Chem..

[9] Woodruff M.A., Hutmacher D.W. (2010). The return of a forgotten polymer-polycaprolactone in the 21st century. Prog. Polym. Sci..

[10] Castillo R.V., Müller A.J., Raquez J.-M., Dubois P. (2010). Crystallization kinetics and morphology of biodegradable double crystalline PLLA-b-PCL diblock copolymers. Macromolecules.

[11] Fortelny I., Ujcic A., Fambri L., Slouf M. (2019). Phase structure, compatibility, and toughness of PLA/PCL blends: A review. Front. Mater..

[12] Song Z.J., Huang X.L., Lu X.L., Lv Q.Q., Xu N., Pang S.J., Pan L.S., Li T. (2018). Improvement of microstructures and properties of poly(lactic acid)/poly(epsilon-caprolactone) blends compatibilized with polyoxymethylene. J. Appl. Polym. Sci..

[13] Ferri J.M., Fenollar O., Jorda-Vilaplana A., Garcia-Sanoguera D., Balart R. (2016). Effect of miscibility on mechanical and thermal properties of poly(lactic acid)/polycaprolactone blends. Polym. Int..

[14] Tsuji H., Yamada T., Suzuki M., Itsuno S. (2003). Blends of aliphatic polyesters. Part 7. Effects of poly(L-lactide-co-epsilon-caprolactone) on morphology, structure, crystallization, and physical properties of blends of poly(L-lactide) and poly(epsilon-caprolactone). Polym. Int..

[15] Muiruri J.K., Liu S., Teo W.S., Kong J., He C. (2017). Highly biodegradable and tough polylactic acid–cellulose nanocrystal composite. ACS Sustain. Chem. Eng..

[16] Ning Z.B., Liu J.J., Jiang N., Gan Z.H. (2017). Enhanced crystallization rate and mechanical properties of poly(l-lactic acid) by stereocomplexation with four-armed poly(ε-caprolactone)-block-poly(d-lactic acid) diblock copolymer. Polym. Int..

[17] Sun Y., Yang L.P., Lu X.H., He C.B. (2015). Biodegradable and renewable poly(lactide)-lignin composites: Synthesis, interface and toughening mechanism. J. Mater. Chem. A.

[18] Ikada Y., Jamshidi K., Tsuji H., Hyon S.H. (1987). Stereocomplex formation between enantiomeric poly(Lactides). Macromolecules.

[19] Schmidt S.C., Hillmyer M.A. (2001). Polylactide stereocomplex crystallites as nucleating agents for isotactic polylactide. J. Polym. Sci. Part B Polym. Phys..

[20] Tsuji H. (2016). Poly(lactic acid) stereocomplexes: A decade of progress. Adv. Drug. Deliv. Rev..

[21] Pan P.J., Liang Z.C., Zhu B., Dong T., Inoue Y. (2009). Blending effects on polymorphic crystallization of poly(L-lactide). Macromolecules.

[22] Shao J., Xiang S., Bian X.C., Sun J.R., Li G., Chen X.S. (2015). Remarkable melting behavior of PLA stereocomplex in linear PLLA/PDLA blends. Ind. Eng. Chem. Res..

[23] Song Y., Wang D.J., Jiang N., Gan Z.H. (2015). Role of PEG segment in stereocomplex crystallization for PLLA/PDLA-b-PEG-b-PDLA blends. ACS Sustain. Chem. Eng..

[24] de Jong S.J., van Dijk-Wolthuis W.N.E., Kettenes-van den Bosch J.J., Schuyl P.J.W., Hennink W.E. (1998). Monodisperse enantiomeric lactic acid oligomers:  Preparation, characterization, and stereocomplex formation. Macromolecules.

[25] Hu J., Wang J.P., Wang M.F., Ozaki Y., Sato H., Zhang J.M. (2019). Investigation of crystallization behavior of asymmetric PLLA/PDLA blend using raman imaging measurement. Polymer.

[26] Iniguez-Franco F., Auras R., Burgess G., Holmes D., Fang X., Rubino M., Soto-Valdez H. (2016). Concurrent solvent induced crystallization and hydrolytic degradation of PLA by water-ethanol solutions. Polymer.

[27] Ning Z.B., Jiang N., Gan Z.H. (2014). Four-armed PCL-b-PDLA diblock copolymer: 1. Synthesis, crystallization and degradation. Polym. Degrad. Stab..

[28] Jing Y.H., Quan C.Y., Liu B., Jiang Q., Zhang C. (2016). A mini review on the functional biomaterials based on poly (lactic acid) stereocomplex. Polym. Rev..

